# E46K Mutant α-Synuclein Is Degraded by Both Proteasome and Macroautophagy Pathway

**DOI:** 10.3390/molecules23112839

**Published:** 2018-11-01

**Authors:** Jia-qing Yan, Yu-he Yuan, Shi-feng Chu, Guo-hui Li, Nai-hong Chen

**Affiliations:** 1 Department of Pharmacy, National Cancer Center/National Clinical Research Center for Cancer, Chinese Academy of Medical Sciences and Peking Union Medical College, Beijing 100021, China; yanjiaqing@cicams.ac.cn (J.-q.Y.); lgh0603@cicams.ac.cn (G.-h.L.); 2 State Key Laboratory of Bioactive Substances and Functions of Natural Medicines, Institute of Materia Medica and Neuroscience Center, Chinese Academy of Medical Sciences and Peking Union Medical College, Beijing 100050, China; yuanyuhe@imm.ac.cn (Y.-h.Y.); chushifeng@imm.ac.cn (S.-f.C.)

**Keywords:** α-synuclein, E46K, proteasome, chaperon-mediated autophagy, macroautophagy

## Abstract

Genetic studies have revealed that rare mutations and multiplications of the gene locus in α-synuclein (α-syn) are implicated in the pathogenesis of Parkinson’s disease (PD). However, the pathological effects of α-syn are still obscure. The neurotoxicity of α-syn is mainly determined by its protein levels, which depend on a balance between synthesis and degradation. Therefore, verifying the possible routes contributing to the clearance of α-syn is important for PD therapy. In this study, we established stable lines overexpressing human wild-type (WT) and E46K mutant α-syn in rat PC12 cells and investigated the degradation pathways of α-syn by using a panel of inhibitors and inducers of lysosome and proteasome function. We also monitored the degradation kinetics of α-syn by using cycloheximide to block protein synthesis. Our data showed that both proteasome and chaperon-mediated autophagy (CMA) are responsible for the degradation of the WT α-syn. Meanwhile, E46K mutant α-syn is mainly degraded by the proteasome and macroautophagy pathway. Compared with the WT protein, E46K mutant α-syn turned over more slowly in PC12 cells. In addition, overexpression of E46K mutant α-syn increased vulnerability of PC12 cells to apoptosis insults when compared with WT α-syn. Our findings may verify the possible routes contributing to the degradation of the E46K mutant α-syn.

## 1. Introduction

Parkinson’s disease (PD) is the second most prevalent neurodegenerative disorder. The primary clinical symptoms of PD are a series of movement deficits such as resting tremor, bradykinesia, and rigidity [1]. The pathological hallmarks of PD are characterized by a selective loss of dopaminergic neurons in the substantia nigra pars compacta as well as the formation of protein inclusions in the surviving neurons called Lewy Bodies (LBs) [2]. The major component of LBs is α-synuclein (α-syn), which is a small protein comprised of 140 amino acid residues with unknown function. Genetic studies have revealed that three points mutations (including A30P, A53T, and E46K) as well as multiplications of the entire gene locus in the gene encoding α-syn lead to familial forms of PD [3], which indicates a pathological role for α-syn in PD etiology. However, the mechanisms underlying the toxicity of α-syn are still poorly understood. It is generally accepted that aberrant aggregation of α-syn plays a pivotal role in the development of PD [4]. The major factor controlling α-syn aggregation is its protein levels, which depend on a balance between synthesis and degradation [5]. Therapeutic strategies to reduce α-syn levels have been demonstrated to be beneficial to PD treatment [6]. Therefore, identifying the precise routes responsible for the degradation of α-syn is critical to PD therapy.

The ubiquitin proteasome system (UPS) and autophagy lysosome pathway (ALP) are the two major proteolytic routes contributing to the clearance of intracellular proteins [7]. UPS is the major pathway responsible for the clearance of short-lived and soluble proteins such as key regulatory proteins that are associated with the cell cycle, signal transduction, apoptosis, and differentiation. In addition, misfolded and damaged proteins are also degraded by the ubiquitin-dependent proteasome pathway [8]. The proteasome degradation is comprised of several sequent steps. First, the ubiquitin-activating enzyme (E1) activates free ubiquitin at the carboxyl end in an ATP-dependent manner and activated ubiquitin is then transferred to the ubiquitin-conjugating enzyme (E2). The ubiquitin-protein ligase (E3) transfers the ubiquitin chains from E2 to lysine residues of the substrate proteins, which serves as a signal for proteasome degradation. Lastly, the 26S proteasome recognizes and degrades the target proteins [9].

Distinct from UPS, ALP is mainly involved in the clearance of long-lived proteins and protein aggregates, which is thought to be an adaptive response under stress conditions such as nutrient deprivation [10]. ALP plays an important role in many physiological and pathological processes such as cancer and neurodegenerative diseases [11,12,13]. According to the ways by which substrates cross into lysosome lumen, there are three types of autophagy including microautophagy, macro-autophagy, and chaperon-mediated autophagy (CMA) [14]. Microautophagy refers to the direct sequestration of cytosolic components by a lysosome. However, its normal functions are still little known. Macroautophagy is considered to be a nonselective degradation process of protein aggregates and organelles [15]. Macroautophagy initiates with the formation of double-membrane vesicles termed autophagosomes, which elongate and engulf substrates. The autophagosomes fuse with lysosome to form autolysosomes in which substrates are degraded [16,17,18,19]. CMA is a selective process involved in the degradation of soluble proteins bearing a KFERQ-like motif. In contrast to microautophagy and macroautophagy, transport of CMA substrate does not require the formation of autophagic vesicles or membrane fusion. Instead, the substrate protein of CMA is first recognized by the heat shock chaperon 70 and delivered to the lysosome surface where it binds to a lysosome associated membrane protein type 2a (LAMP2A), which is the only known protein associated with CMA. With the help of LAMP2A, a substrate protein is translocated into lysosome lumen and rapidly degrades [20].

Recent publications have suggested that both proteasome and lysosome pathway are associated with the clearance of wild-type (WT) and mutant forms of α-syn (A30P and A53T) [21,22]. However, the precise degradation pathways for E46K mutant α-syn are still unclear. In this study, we established stable lines for human WT and E46K mutant α-syn in PC12 cells and compared the degradation pathways and kinetics of the two proteins. We discovered that WT α-syn was degraded by both proteasome and CMA pathway while E46K mutant α-syn was mainly cleared through a proteasome and a macro-autophagy pathway in PC12 cells. We also found that E46K mutant α-syn turned over more slowly when compared with WT α-syn. Furthermore, cells overexpressing E46K mutant α-syn exhibited enhanced susceptibility to toxicity insults when compared with the WT protein. These findings may not only help us better understand the pathological roles of mutant α-syn but also provide us with some therapeutic implications for PD treatment.

## 2. Results

### 2.1. Establishment of PC12 Cell Stably Overexpressing Human WT and E46K Mutant α-syn

**Figure 1:**
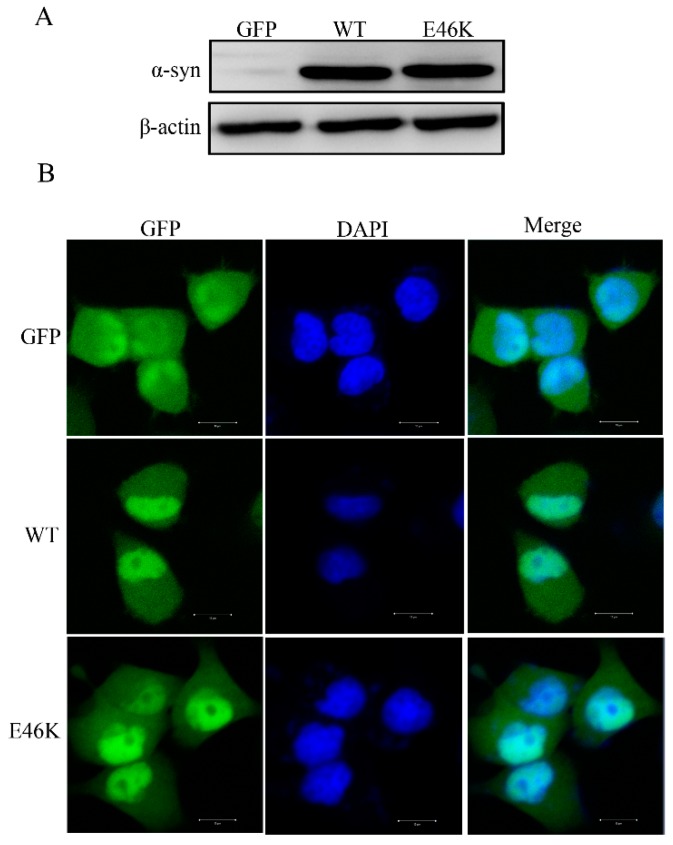
Establishment of PC12 cell stably overexpressing human WT and E46K mutant α-syn. (**A**) Western blotting analysis of the expression of human WT and E46K mutant α-syn. PC12 cells were transfected with pEGFP-N1-α-syn, pEGFP-N1-E46K mutant α-syn, or an empty vector using Lipofectamine 2000, according to the manufacturer’s instructions. Poly-colonies were selected in the presence of 800 μg/mL G418. Cells were lysed and extracts were subjected to immunoblot analysis using an anti-α-syn antibody. (**B**) Immunofluorescence images of stable PC12 cells expressing E46K mutant α-syn. Scale bar: 10 mm.

We established stable lines for human WT and E46K mutant α-synuclein in PC12 cells and assessed α-syn expression by Western blotting using an anti-α-syn antibody. As expected, we detected a dramatic expression of WT and E46K mutant α-syn and compared it with the empty vector group (Figure 1A). We next observed the distribution of WT and E46K mutant α-syn in PC12 cells. Immunofluorescence studies showed that, when compared with GFP, which was evenly distributed across the cell, both WT and E46K mutant α-syn were distributed across the cell with a nuclear localization (Figure 1B). This was similar to the previous report [23]. In addition, we cannot observe any significant aggregation of either WT or E46K mutant α-syn in our stable cell lines (Figure 1B).

### 2.2. E46K Mutant α-syn Was Degraded by Proteasome and Macroautophagy Pathway

**Figure 2:**
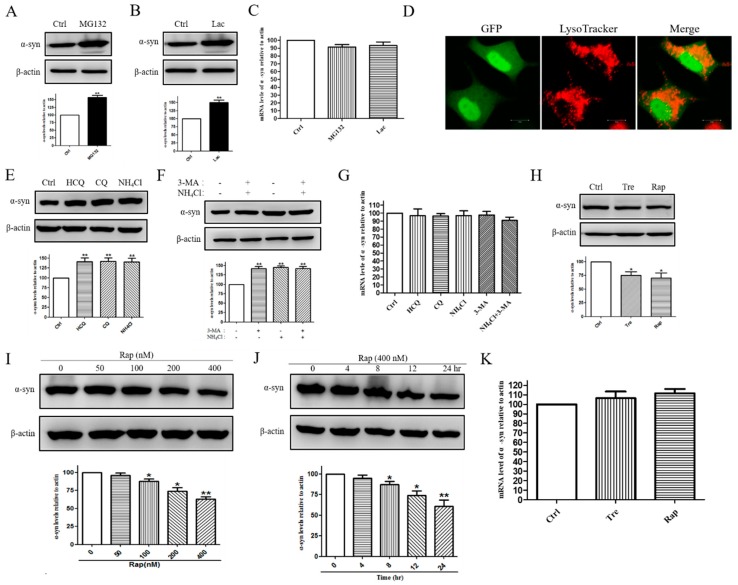
The E46K mutant α-syn is degraded by a proteasome and a macro-autophagy pathway. (**A**) PC12 cells stably overexpressing the E46K mutant α-syn were treated with 10 μM MG132 for 24 h. Cells were lysed and subjected to SDS-PAGE using an anti-α-syn antibody. (**B**) Stable PC12 cells overexpressing E46K mutant α-syn were treated with 10 μM Lac for 24 h. α-Syn levels were assessed by using an anti-α-syn antibody. (**C**) Cells were treated with 10 μM MG132 or 10 μM Lac for 24 h and mRNA levels of the E46K mutant α-syn were detected by using RT-PCR. (**D**) Cells were labeled with Lyso-Tracker for 20 min and visualized by using a confocal microscopy. Representative images were shown. Scale bar: 10 mm. (**E**) Cells were treated with 100 μM CQ, 30 μg/mL HCQ, and 20 mM NH_4_Cl for 24 h. Cells were lysed and subjected to SDS-PAGE using an anti-α-syn antibody. (**F**) Cells were incubated with 10 mM 3-MA, 20 mM NH_4_Cl, and a combination of 3-MA and NH_4_Cl for 24 h. Cells were lysed and subjected to immunoblot analysis using an anti-α-syn antibody. (**G**) Cells were treated with 100 μM CQ, 30 μg/mL HCQ, 20 mM NH_4_Cl, 10 mM 3-MA, and a combination of NH_4_Cl with 3-MA for 24 h. RT-PCR was conducted to assess the mRNA levels of E46K mutant α-syn. (**H**) PC12 cells were treated with 100 mM Tre and 200 nM Rap for 24 h and α-syn levels were assessed using Western blotting. (**I**) PC12 cells were treated with Rap for indicated concentration and harvested for immunoblotting for α-syn. (**J**) PC12 cells were treated with 400 nM Rap for the indicated periods of time and cell extracts were subjected to the immunoblot. Representative blots of three independent experiments were shown. β-actin levels demonstrate equal loading. (**K**) Cells were treated with 100 mM Tre of 400 nM Rap for 24 h and mRNA levels of E46K mutant α-syn were detected by RT-PCR. Data were expressed as means ± SD for three independent experiments performed in triplicate. * *p* < 0.05, ** *p* < 0.01 compared with the control group.

In order to identify the role for the proteasome in the degradation of the E46K mutant α-syn, we treated cells with the proteasome inhibitor MG132 for 24 h and measured α-syn levels by Western blotting. Our results showed that inhibition of proteasome activity by MG132 led to a clear increase in the levels of the E46K mutant α-syn when compared with the control group (*p* < 0.01, Figure 2A). Consistent with this, treatment with another proteasome inhibitor lactacystin (Lac) for 24 h also significantly increased an E46K mutant α-syn levels by (49.95 ± 7.74)% was compared with the control group (*p* < 0.01, Figure 2B). An increased protein level may be due to increased expression or decreased degradation so the RT-PCR was conducted to investigate the influence of the two inhibitors on the mRNA level of E46K mutant α-syn. As shown in Figure 2C, treatment with MG132 or Lac did not increase E46K mutant α-syn expression. These data suggested that ubiquitin-dependent proteasome pathway was involved in the degradation of E46K mutant α-syn. We next focused our attention on whether the E46K mutant α-syn was also degraded by a lysosome-dependent degradation pathway. We first performed the immunofluorescence analysis to observe the cellular distribution of the E46K mutant α-syn in PC12 cells. Confocal studies showed that E46K mutant α-syn was localized inside acidic vacuoles and stained by using LysoTracker (Figure 2D), which labels acid vacuoles such as lysosomes and degradative autophagic vacuoles. This indicates a role for ALP in the degradation of the E46K mutant α-syn. On the other hand, inhibition of lysosome function by hydroxychloroquine (HCQ), chloroquine (CQ), and NH_4_Cl resulted in a dramatic elevation of E46K mutant α-syn levels by (40.99 ± 9.69, 42.23 ± 8.91, and 40.43 ± 9.66) percent, respectively, (*p* < 0.01, Figure 2E). These findings indicated that the E46K mutant α-syn was degraded through the lysosome pathway. As mentioned above, ALP consists of microautophagy, CMA, and macroautophagy. We next proceeded to identify the precise lysosomal routes contributing to E46K mutant α-syn turnover. We treated cells with a selective macroautophagy inhibitor 3-methyladenine (3-MA) and a lysosome inhibitor NH_4_Cl for 24 h and followed E46K mutant α-syn levels by Western blotting. We found that both 3-MA and NH_4_Cl treatment led to a marked increase in the levels of E46K mutant α-syn when compared with the control group (*p* < 0.01, Figure 2F). Additionally, 3-MA increased E46K mutant α-syn levels to an extent similar with that of NH_4_Cl (Figure 2F). In addition, we also combined NH_4_Cl and 3-MA to ensure that the maximum inhibition of the lysosome pathway was attained (Figure 2F). As shown in Figure 2G, treatment with autophagy-lysosome system inhibitors did not increase E46K mutant α-syn expression. These data led us to the hypothesis that the E46K mutant α-syn was degraded by the macroautophagy pathway. This assumption was further validated by the following experiments. Induction of macroautophagy by rapamycin (Rap) and trehalose (Tre) could significantly reduce E46K mutant α-syn levels by (24.73 ± 6.64 and 29.67 ± 9.34) percent, respectively (*p* < 0.05, Figure 2H). Furthermore, Rap promoted E46K mutant α-syn degradation in a concentration and time-dependent manner (Figure 2I,J). Additionally, treatment with these agents did not affect mRNA levels of α-syn (Figure 2K). Together, these data suggested that the E46K mutant α-syn was degraded by both the proteasome and the macro-autophagy pathway in PC12 cells.

### 2.3. WT α-syn Was Degraded by Proteasome and CMA Pathway

**Figure 3:**
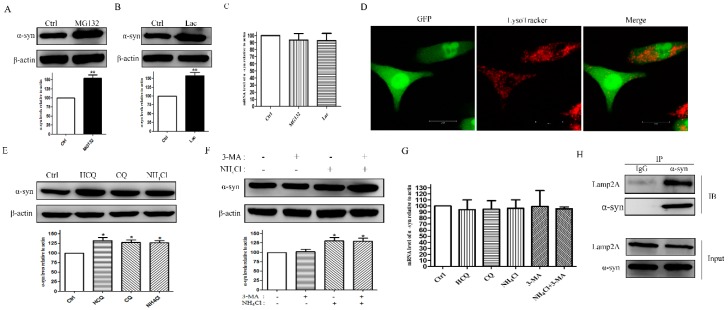
WT α-syn was degraded by the proteasome and the CMA pathway. PC12 cells stably overexpressing WT α-syn were treated with 10 μM MG132 (**A**) or 10 μM Lac (**B**) for 24 h. Cells were lysed and subjected to SDS-PAGE by using an anti-α-syn antibody. (**C**) Cells were treated with 10 μM MG132 or 10 μM Lac for 24 h and mRNA levels of WT α-syn were detected by using the RT-PCR. (**D**) Cells were labeled with a Lyso-Tracker for 20 min and was visualized by using a confocal microscopy. Representative images were shown. Scale bar: 10 mm. (**E**) Cells were treated with 100 μM CQ, 30 mg/mL HCQ, and 20 mM NH_4_Cl for 24 h. Cells were harvested and subjected to SDS-PAGE using an anti-α-syn antibody. (**F**) Cells were treated with 10 mM 3-MA and 20 mM NH_4_Cl for 24 h. α-Syn levels were determined by using Western blotting analysis. (**G**) Cells were treated with 100 μM CQ, 30 μg/mL HCQ, 20 mM NH_4_Cl, 10 mM 3-MA, and a combination of NH_4_Cl with 3-MA for 24 h. RT-PCR was conducted to assess the mRNA levels of WT α-syn. (**H**) Lysates from PC12 cells were immuno-precipitated with an anti-α-syn antibody and blotted with an anti-LAMP2A antibody. IgG was used as a negative control. Representative blots of three independent experiments were shown. β-Actin levels demonstrated equal loading. Data were expressed as means ± SD for three independent experiments performed in triplicate. * *p* < 0.05, ** *p* < 0.01 when compared with the control group.

We then investigated the possible routes for the degradation of WT α-syn. Western blotting results showed that treatment with proteasome inhibitors MG132 and Lac for 24 h could dramatically elevate WT α-syn levels by (54.27 ± 9.46 and 57.43 ± 9.48) percent when compared with the control group. (*p* < 0.01, Figure 3A,B), which indicates a role for proteasomes in the degradation of WT α-syn. RT-PCR was also conducted to investigated the effects on mRNA levels of WT α-syn upon treatment with proteasome inhibitors. We did not observe significant changes, which is shown in Figure 3C. We also observed that WT α-syn localized within acidic vacuoles were labeled with a LysoTracker (Figure 3D). Consistent with this, lysosome inhibition by HCQ, CQ, and NH_4_Cl significantly increased WT α-syn levels by (32.36 ± 7.91, 27.69 ± 6.78, 26.90 ± 5.84) percent, respectively (*p* < 0.05, Figure 3E). Next, we treated cells with the selective macroautophagy inhibitor 3-MA and lysosome inhibitor NH_4_Cl as well as a combination of the two drugs for 24 h and followed α-syn degradation by using Western blotting. Our results showed that lysosome inhibition led to a clear accumulation of WT α-syn by (30.98 ± 8.37) percent when compared with a control group (*p* < 0.05, Figure 3F). In contrast, we failed to observe any significant changes in α-syn levels under treatment of 3-MA (Figure 3F). Meanwhile, inhibiting the autophagy lysosome pathway activity did not increase WT α-syn expression (Figure 3G). These data suggested that WT α-syn might be degraded by CMA. Since α-syn contains a 95VKKDQ99 sequence, which is consistent with the CMA recognition motif, we then preformed co-immunoprecipitation assays to detect the interaction between α-syn and LAMP2A. Our data showed that α-syn co-immunoprecipitated with LAMP2A in PC12 cells (Figure 3H), which further confirmed our hypothesis that α-syn was a CMA substrate. Collectively, our data indicated that WT α-syn was degraded by the proteasome and CMA pathway.

### 2.4. E46K Mutant α-syn Turned over More Slowly Compared with WT α-syn

**Figure 4:**
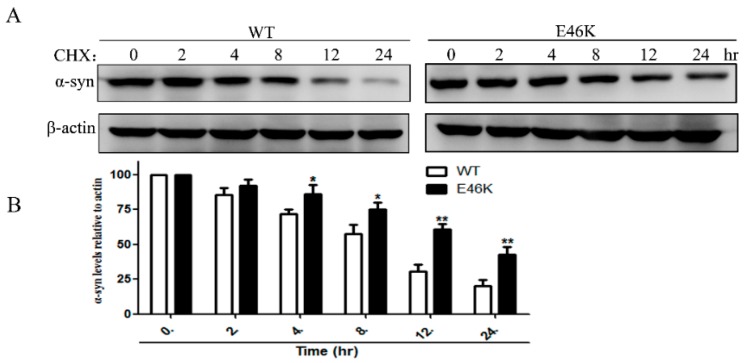
E46K mutant α-syn turned over more slowly when compared with WT α-syn. (**A**) PC12 cells stably overexpressing WT and E46K mutant α-syn were treated with 10 mg/mL CHX for the indicated periods of time and α-syn levels were measured by Western blotting. (**B**) Representative blots of three independent experiments were shown. β-Actin levels demonstrated equal loading. Data were expressed as means ± SD for three independent experiments performed in triplicate. * *p* < 0.05, ** *p* < 0.01 when compared with the WT group.

We then monitored the degradation kinetics of WT and E46K mutant α-syn in PC12 cells. Cycloheximide (CHX) was used to block protein synthesis for indicated periods of time and α-syn degradation was assessed by Western blotting. Our data showed that the E46K mutant α-syn turned over more slowly when compared with that of WT α-syn after protein synthesis was stopped (Figure 4).

### 2.5. Cells Overexpressing E46K Mutant α-syn Exhibited Enhanced Vulnerability to Apoptosis Insults

**Figure 5:**
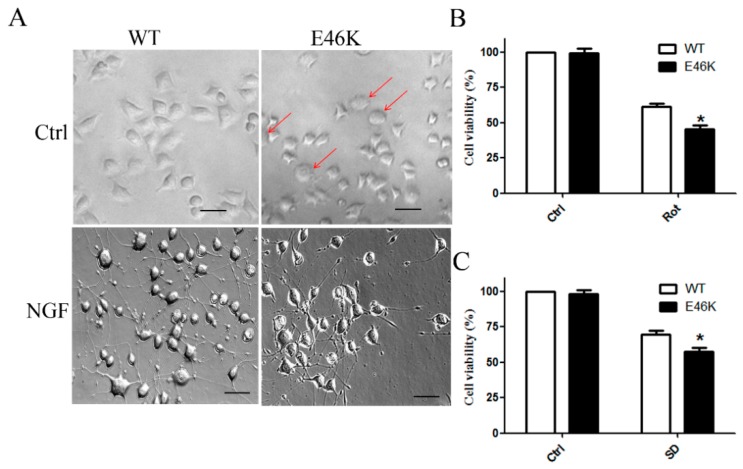
Cells overexpressing E46K mutant α-syn exhibited enhanced vulnerability to apoptosis insults. (**A**) Morphological changes of PC12 cells overexpressing WT and E46K mutant α-syn were observed. The arrowhead indicated abnormal cells. Scale bar: 50 μm. (**B**) PC12 cells overexpressing WT and E46K mutant α-syn were treated with 10 μM Rot for 24 h and cell viability was assessed by the MTT assay. (**C**) PC12 cells overexpressing WT and E46K mutant α-syn were treated with serum-free medium for 24 h and cell viability was assessed by the MTT assay. Data were expressed as means ± SD for three independent experiments performed in triplicate. * *p* < 0.05 when compared with the WT group.

Lastly, we compared the toxicity of the two proteins. Our data showed that PC12 cells overexpressing the E46K mutant α-syn exhibited a number of morphological changes such as an increased size when compared with cells transfected with the WT α-syn. After treatment with a 100-ng/mL nerve growth factor (NGF) for seven days, cells overexpressing WT α-syn established an integrate neuritic network. In contrast, cells overexpressing the E46K mutant α-syn showed a limited response to NGF treatment (Figure 5A). We also found that transfection of the E46K mutant α-syn showed no effects on cell viability with WT cells but showed decreased cell viability under rotenone (Rot) treatment (Figure 5B) and serum deprivation (SD) (Figure 5C). These findings suggested that overexpression of E46K mutant α-syn increased the vulnerability of PC12 cells to toxic insults when compared with the WT protein.

## 3. Discussion

Since the discovery that α-syn is the major component existing in the LBs as well as mutations in the gene encoding α-syn causes familial forms of PD, this protein has attracted much attention. It is thought to play a role in the regulating dopamine release, which facilitates synaptic plasticity and promotes a SNARE complex assembly [24,25,26]. Accumulation publications have placed α-syn as the central player in the pathogenesis of PD [27]. Although the potential mechanisms underlying the toxic effects of α-syn are still poorly understood, it is generally accepted that intracellular protein levels of α-syn determine its toxicity and therapeutic strategies to reduce its protein levels, which will help to ameliorate its toxicity and prevent neurodegeneration. Therefore, it is critical for exploring the degradation pathway for the α-syn turnover.

In this study, we investigated the possible routes for the degradation of WT and the E46K mutant α-syn in PC12 cells. Our data showed that E46K mutant α-syn was degraded by proteasome and macroautophagy pathway while WT α-syn was mainly cleared through the proteasome and CMA pathway. Furthermore, compared with WT α-syn, E46K mutant α-syn turned over more slowly.

Our findings suggested that both WT and E46K mutant α-syn can be degraded by the ubiquitin-dependent proteasome pathway, which indicates a central role for the proteasome in α-syn turn over. Compared with UPS, the role of ALP in α-syn degradation is diverse. Our data showed that both the WT and the E46K mutant α-syn localized within acidic vacuoles, which represent lysosomes and degradative autophagic vacuoles and inhibit lysosome function significantly, promoted the accumulation of both WT and E46K mutant α-syn, which indicates a role for ALP in the degradation of the WT and E46K mutant α-syn. Further study showed that selective inhibition of macroautophagy led to a marked increase in the levels of the E46K mutant α-syn similar with that of lysosome inhibition. In contrast, no obvious changes in WT α-syn levels were observed under macroautophagy inhibition. This led us to the hypothesis that the E46K mutant α-syn was degraded by macroautophagy while WT α-syn was turned over via CMA. This assumption was further confirmed by the following assays. Induction of macroautophagy by Rap and Tre dramatically accelerated the E46K mutant α-syn clearance. On the other hand, co-immuno-precipitate analysis demonstrated the association between WT α-syn and LAMP2A. Previous studies have demonstrated that WT α-syn was degraded by both CMA and proteasome pathway [21,23]. This was consistent with our study. It has also been reported that the A30P and A53T mutant α-syn are also degraded by both proteasome and lysosome pathway. Compared with the A30P mutant protein, the A53T mutant α-syn was more sensitive to macro-autophagy degradation, which may be due to its greater propensity to aggregate [23]. The factors regulating WT and mutant α-syn degradation remains unclear. Proteasome and CMA mainly degrade small soluble proteins while macro-autophagy is the major pathway for the degradation of protein aggregates as well as damaged organelles. Under physiological conditions, α-syn exists as a soluble protein with random coil conformation. Mutations in α-syn change its conformation and promote aggregation [28,29]. However, we did not observe any significant aggregation of the E46K mutant α-syn in our cell lines. Whether the differences between WT and mutant α-syn was due to different conformations between the two proteins needs to be further studied.

We also compared the degradation kinetics of the WT and the E46K mutant α-syn in our stable cell lines. Our data showed that, after inhibiting protein synthesis by CHX, E46K mutant α-syn turned over more slowly when compared with the WT protein.

Emerging evidence has implicated a causal role for UPS and ALP dysfunction in the pathogenesis of PD. It has been reported that mutations in Parkin and ubiquitin carboxy-terminal hydrolase L1 in which both are involved in ubiquitin-dependent degradation were associated with inherited PD [30,31]. The link between UPS dysfunction and PD was further validated by the findings that proteasome inhibition led to neurodegeneration and LBs formation [32]. On the other hand, deregulation of autophagy is considered to play a critical role in the pathogenesis of PD [33,34,35]. Furthermore, impaired CMA leads to a loss of dopaminergic neurons in rats [36]. Dysfunction of the proteolytic pathways leads to accumulation of α-syn due to inefficient clearance. In turn, increased α-syn may further impair UPS and ALP functions. It is reported that A30P and A53T mutant α-syn impair the CMA function and decrease proteasome activity [37]. In our previous study, overexpression of the E46K mutant α-syn inhibits macro-autophagy at an early stage of autophagosome formation, which leads to an impaired UPS function [38]. These findings together with our previous discoveries generate a reciprocal relationship between the E46K mutant α-syn and the protein degradation system. Our findings may help better understand the molecular pathogenesis of PD.

Accumulation evidence has suggested that the E46K mutation in α-syn increased the risk of PD. Expression of the E46K mutant α-syn in rats induces early PD features and enhances vulnerability to mitochondrial impairment [39]. In our study, we found that overexpression of E46K mutant α-syn exhibited enhanced toxicity than the WT protein. No apparent differences were observed in cell viability between cells transfected with the E46K mutant and the WT α-syn. However, cells overexpressing the E46K mutant α-syn displayed enhanced vulnerability to apoptosis insults such as Rot treatment and serum deprivation. The toxicity of the α-syn is associated with its protein levels. Our findings that the E46K mutant α-syn turned over slowly compared with WT α-syn could partly explain how mutant forms of α-syn exhibit enhanced toxicity than the WT protein. However, whether this difference is due to the distinct degradation pathways between WT and the E46K mutant α-syn is still elucidated.

Although the mechanisms that decide whether α-syn will be degraded by the UPS or ALP are still unclear, future therapeutic applications targeting the specific degradation pathways may be applied to PD treatment [40,41]. It has been reported that both of the autophagy activators rapamycin and trehalose were demonstrated to be potential therapeutic approaches for neurodegenerative diseases [42,43]. In this study, we found that both rapamycin and trehalose showed some potential effects on the E46K mutant α-syn clearance. Such therapies may not only reduce α-syn levels but also alleviate its adverse effects on protein degradation pathways. Therefore, our study may provide some implications for PD therapy.

## 4. Materials and Methods

### 4.1. Cell Culture

Rat pheochromocytoma (PC12) cells (Peking Union Medical College, Beijing, China) were maintained in Dulbecco’s modified Eagle’s medium (Invitrogen, Carlsbad, CA, USA) supplemented with 5% fetal bovine serum (Invitrogen), 10% horse serum (Invitrogen), 100 U/mL penicillin, 100 μg/mL streptomycin, and 2 mM l-glutamine. Cells were maintained at 37 °C in a humidified atmosphere with 5% CO_2_. Media were replaced every two or three days and cells were passaged twice per week.

### 4.2. Generation of PC12 Cells Stably Overexpressing Human WT and E46K Mutant α-Syn

pEGFP-N1 plasmid expressing human WT syn was our laboratory stock prepared as described previously [44]. The E46K mutation was induced by PCR-based mutagenesis and sub-cloned in the XhoI-HindIII sites of a pEGFP-N1 vector, which was previously reported [45]. All constructs were confirmed by sequencing. PC12 cells were transfected with pEGFP-N1-syn, pEGFP-N1-E46K mutant α-syn, and an empty vector using Lipofectamine 2000, according to the manufacturer’s instructions. Positive colonies were selected in the presence of 800 μg/mL G418 (Amresco, Solon, OH, USA).

### 4.3. Drug Treatment

For drug treatment, PC12 cells overexpressing WT or E46K mutant α-syn were incubated in growth media containing either 10 μM MG132 (Sigma, St. Louis, MO, USA), 10 μM Lac (Sigma), 100 μM CQ (Sigma), 30 μg/mL HCQ (Sigma), 20 mM NH_4_Cl, 10 mM 3-MA (Sigma), and 100 mM Tre (Sigma) for 24 h. CHX (Sigma) was used at 10 μg/mL for the indicated periods of time. Rap (Sigma) was used at the indicated concentrations and periods of time.

### 4.4. Western Blot Analysis

Cells were treated as described above and lysed in lysis buffer containing 50 mM Tris-HCl, 150 mM NaCl, 1% NP-40, 1 mM EDTA, and supplemented with the protease inhibitor cocktail (Sigma) for 30 min on ice. Protein contents were determined by the BCA assay. Equal amounts of proteins for each sample were separated by the SDS-PAGE gel and then transferred to the PVDF membrane (Millipore, Billerica, MA, USA). The membrane was blocked with 3% BSA (Sigma) for 2 h at room temperature and incubated with the following primary antibodies: anti-α-syn antibody (1:500 dilutions from Santa Cruz Biotechnology, Santa Cruz, CA, USA) and anti-β-actin antibody (1:1000 dilutions from Sigma) overnight at 4 °C. Blots were labeled with anti-rabbit or anti-mouse horseradish peroxidase (HRP)-conjugated second antibodies (All 1:5000 dilutions from KPL, Gaithersburg, MD, USA) for 1 h at room temperature. Bands were detected by enhanced chemiluminescence (ECL). Images were analyzed using Gel-Pro Analyzer software (Media Cybernetics, Silver Spring, MD, USA).

### 4.5. Immunofluorescence Staining

Cells were grown on coverslips coated with 0.01% poly-l-lysine (Sigma) and then fixed in 4% paraformaldehyde for 20 min at room temperature. After washed three times in PBS, cells were permeabilized in 0.1% Triton-X-100 (Sigma) for 10 min and blocked in 3% goat serum for 1 h at room temperature. Images were acquired by using a confocal microscope.

### 4.6. Staining for Lysosomes

Cells were grown in confocal dishes coated with 0.01% poly-l-lysine. After incubated with Lyso Tracker Red ND99 (Invitrogen) at 50 nM for 20 min at 37 °C, cells were washed three times using PBS and visualized with a confocal microscope.

### 4.7. RT-PCR

Total RNA was extracted from PC12 cells, which stably overexpressed WT or E46K mutant α-synuclein by using Trizol (Invitrogen). 1 μg RNA of each sample was reverse transcribed to cDNA by using the Reverse Transcription Kit (Transgen Biotech, Beijing, China), according to the manufacturer’s instructions. The indicated genes mRNA expression was analyzed by using KAPA SYBR FAST qPCR Master Mix Kit (Kapa Biosystems, Johannesburg, South Africa) with primers for α-synuclein (5′-gagctgaagggcatcgactt; 5′-gacaagcagaagaacggcat) and for β-actin (5′-cacccgcgagtacaaccttc; 5′-cccatacccaccatcacacc), respectively. RT-PCR was performed by the following parameters: 3 min at 95 °C, 3 s at 95 °C, 25 s at 60 °C, 40 cycles of amplification was needed.

### 4.8. Co-Immunoprecipitation Assay

PC12 cells were lysed in IP buffer (25 mM Tris, pH 7.4, 150 mM NaCl, 1 mM EDTA, 1% NP-40, 5% glycerol with complete protease inhibitor cocktail) for 30 min on ice followed by centrifugation for 30 min at 13,000 rpm. A small fraction of the lysates was stored as inputs. In addition, 500 μg protein of each lysate was incubated for 4 h at 4 °C with anti-α-syn antibodies and for another 2 h with protein A-coupled magnetic beads (Invitrogen). After incubation, beads were washed three times in IP buffer and boiled in 2 × SDS-PAGE buffer for 10 min. IP products were subjected to SDS-PAGE electrophoresis and immunoblotted for LAMP2A (1:500 dilutions from Santa Cruz) together with the inputs.

### 4.9. Morphological Observation and MTT Assay

PC12 cells overexpressing WT or E46K mutant α-syn were differentiated with 100 ng/mL NGF (Sigma) for 7 days and morphological changes were observed by using a phase-contrast microscopy (Olympus, Tokyo, Japan). Cell viability was assessed by the MTT assay. Cells were treated with and without 10 μM rotenone (Sigma) or serum-free medium for 24 h and then incubated with 10 μL MTT (Sigma) at 5 mg/mL for 4 h at 37 °C and the medium was carefully removed. The formazan crystals were dissolved in 100 μL DMSO (Sigma) and absorbance was measured by using a Microplate Reader (Thermo Fisher Scientific, Waltham, MA, USA).

### 4.10. Statistical Analyses

Data were expressed as means ± SD. Significant differences between different groups were assessed by a Student’s *t*-test or One-Way ANOVA using GraphPad Prism 5 software (La Jolla, CA, USA). The difference was determined to be significant if the *p* value was <0.05.
